# Letter to the editor regarding the comment by Gupta and Goswami about our article “individual and household risk factors of severe acute malnutrition among under-five children in Mao, Chad: a matched case-control study”

**DOI:** 10.1186/s13690-019-0344-2

**Published:** 2019-03-28

**Authors:** Jovana Dodos, Chiara Altare, Mathias Altmann

**Affiliations:** Action contre la Faim, 14/16 Boulevard Douaumont - CS 80060, 75854 Paris, CEDEX 17 France

**Keywords:** Severe acute malnutrition, SAM, Children 6–59 months, Childhood illnesses, Environmental determinants, Risk factors, Matched case-control study

## Abstract

We would like to thank the authors Sunanda Gupta and Kiran Goswami for their interest in our article “Individual and household risk factors of severe acute malnutrition among under-five children in Mao, Chad: a matched case-control study”. In this response we aim to address their criticisms.

First of all, while we recognize that our title could have specified the precise age of the study population, this would have not added much to the title itself. Most importantly this information is clearly stated in the methodology of the article.

Anthropometric measurements at the hospital were conducted with a wooden height board and salter scale, which are standard equipment in government run health centers in poor resource settings like Chad. Study teams used a similar wooden height board and an electronic scale for measurement of the controls. While the scales used for cases and controls differ in terms of precision, we deem the possible bias to be minor. The worst scenario would imply that children who are at the hedge between severe and moderate malnutrition might have been considered as SAM. We think this would not compromise the results of our study. Adapting to local conditions is a necessity when conducting operational research in poor resource setting.

We decided to identify vaccination status of children based only on vaccination cards, and not on caregiver’s recall, to reduce the risk of inaccuracy. There is some evidence that household vaccination information may not be reliable [1].

We did not specify but we did back-translation of our questionnaire as this is a standardized procedure for this type of studies.

Gupta et al. affirm MUAC is not adapted to study nutritional status for adult and recommend the use of BMI. This is incorrect. MUAC has been reported to correlate closely with BMI and is a much simpler alternative for field conditions to detect adult undernutrition [2, 3]. MUAC is furthermore a more recommendable measure to use in clinical practice for assessing poor nutritional status [4], and a better predictor of mortality [5]. Cut-offs values have been internationally defined [6]. Lastly, differently from BMI, MUAC can be used also for pregnant women [7].

The sample size calculation was done using EpiInfo® (version 7.2.2.6.) for a matched case-control study. We think some differences in sample size calculation can be observed depending on the software and/or the test used. Our sample size allowed to detect risk factors with an OR of 3 and a prevalence of 80% among controls. However, the OR can be lower if the prevalence among controls is lower. We clearly stated in out article that “*Resources allowed to increase the sample size to 411, including 137 cases and 274 controls. Such an increment in sample size augmented the power of the study.”* So the difference between the two calculations is not because of 10% loss due to non-response as Gupta et al. mentioned.

Gupta et al. were not clear if more than one child from the same family had been taken as cases or controls. In our article we explicitly mentioned that “*Controls were recruited from the same neighborhood where cases reside. After completing the interview with the case caretaker, data collectors visited the closest neighboring households to identify eligible children for the controls*.” Thus, the controls were not selected from the same households neither from the same family.

Gupta et al. feel that one single model, including household and individual risk factors, would be better to study the risk factors. What happens when we have all variables (individual + household) in one model is that risk factors at one level can mask risk factors at another level. We have seen, for example, that recent diarrhoea masks poor household sanitation. This does not mean that the two risk factors are not on the causal pathway. Having models at two levels gives us some idea of the causal pathway (we fill in the links with biological plausibility) and yields much more information. We believe this approach better corresponds to the purpose of our study i.e. identify intervention targets and allow more effective design and prioritization of humanitarian interventions. Furthermore, putting all variables in one model raises the issue of sparse data bias [8], due to the fact that most cases had comorbidities at admission (diarrhoea, fever, vomiting, cough), excluding the possibility to introduce other risk factors in the model. Large confidence intervals might be due to this sparse data bias, and not due to low number of cases.

Gupta et al. suggested to use a *p*-value of 0.2 rather than 0.05 for the stepwise selection procedure. This would be true for a forward elimination, but we used a backward elimination. We first put all variables in the model and keep, as recommended, only those with a p-value < 0.05 in the final model.

Gupta et al. did understand that cases were interviewed at the hospital, leading to a differential recall bias. However, only morbidity data was collected at the hospital - anthropometric measures were extracted from the hospital register. As we started in our article, “*data on health and nutritional status of caretaker, household WASH (water, sanitation and hygiene) conditions, socio-economic and demographic data were collected on the following day during the household visit through face-to-face interview*”, for both groups. Thus we still believe that the risk for recall and social desirability bias if any is similar for both groups.

With regard to complementary feeding practices, we acknowledged that additional information on type, preparation, consistency of the family dish could have provided insights on the mechanism linking it with undernutrition. However, this was beyond the scope of our study. We did not criticize the feeding of children with a family dish, but simply highlighted the risks it can possibly carry.

We believe the issues raised by Gupta et al. are minor and do not alter in any way the conclusions and the validity of our study.

## Abbreviations

BMI: Body Mass Index
MUAC: Mid Upper Arm Circumference
OR: Odds Ratio
SAM: Severe Acute Malnutrition
WASH: Water, Sanitation and Hygiene
